# Impaired neuronal KCC2 function by biallelic *SLC12A5* mutations in migrating focal seizures and severe developmental delay

**DOI:** 10.1038/srep30072

**Published:** 2016-07-20

**Authors:** Hirotomo Saitsu, Miho Watanabe, Tenpei Akita, Chihiro Ohba, Kenji Sugai, Winnie Peitee Ong, Hideaki Shiraishi, Shota Yuasa, Hiroshi Matsumoto, Khoo Teik Beng, Shinji Saitoh, Satoko Miyatake, Mitsuko Nakashima, Noriko Miyake, Mitsuhiro Kato, Atsuo Fukuda, Naomichi Matsumoto

**Affiliations:** 1 Department of Human Genetics, Graduate School of Medicine, Yokohama City University, 3-9 Fukuura, Yokohama 236-0004, Japan; 2 Department of Neurophysiology, Hamamatsu University School of Medicine, 1-20-1 Handayama, Hamamatsu 431-3192, Japan; 3 Department of Child Neurology, National Center Hospital, National Center of Neurology and Psychiatry, 4-1-1 Ogawahigashi-cho, Kodaira, Tokyo 187-8551, Japan; 4 Department of Genetics, Hospital Kuala Lumpur, Jalan Pahang, Kuala Lumpur 50586, Malaysia; 5 Department of Pediatrics, Hokkaido University Graduate School of Medicine, North 15 West 7, Sapporo 060-8638, Japan; 6 Department of Pediatrics, National Defense Medical College, 3-2 Namiki, Tokorozawa, Saitama 359-8513, Japan; 7 Department of Pediatrics, Institute of Pediatrics, Hospital Kuala Lumpur, Jalan Pahang, Kuala Lumpur 50586, Malaysia; 8 Department of Pediatrics and Neonatology, Nagoya City University Graduate School of Medical Sciences, 1 Kawasumi, Mizuho-Cho, Nagoya 467-8601, Japan; 9 Department of Pediatrics, Showa University School of Medicine, 1-5-8 Hatanodai, Tokyo 142-8666, Japan

## Abstract

Epilepsy of infancy with migrating focal seizures (EIMFS) is one of the early-onset epileptic syndromes characterized by migrating polymorphous focal seizures. Whole exome sequencing (WES) in ten sporadic and one familial case of EIMFS revealed compound heterozygous *SLC12A5* (encoding the neuronal K^+^-Cl^−^ co-transporter KCC2) mutations in two families: c.279 + 1G > C causing skipping of exon 3 in the transcript (p.E50_Q93del) and c.572 C >T (p.A191V) in individuals 1 and 2, and c.967T > C (p.S323P) and c.1243 A > G (p.M415V) in individual 3. Another patient (individual 4) with migrating multifocal seizures and compound heterozygous mutations [c.953G > C (p.W318S) and c.2242_2244del (p.S748del)] was identified by searching WES data from 526 patients and *SLC12A5-*targeted resequencing data from 141 patients with infantile epilepsy. Gramicidin-perforated patch-clamp analysis demonstrated strongly suppressed Cl^−^ extrusion function of E50_Q93del and M415V mutants, with mildly impaired function of A191V and S323P mutants. Cell surface expression levels of these KCC2 mutants were similar to wildtype KCC2. Heterologous expression of two KCC2 mutants, mimicking the patient status, produced a significantly greater intracellular Cl^−^ level than with wildtype KCC2, but less than without KCC2. These data clearly demonstrated that partially disrupted neuronal Cl^−^ extrusion, mediated by two types of differentially impaired KCC2 mutant in an individual, causes EIMFS.

Epilepsy of infancy with migrating focal seizures (EIMFS) (also known as migrating partial seizures in infancy) is one of the electroclinical syndromes characterized by migrating polymorphous focal seizures that start within the first 6 months of life and are followed by progressive deterioration of psychomotor development^1^. Mutations in several genes [*KCNT1*, *SCN1A*, *SCN2A*, *SCN8A*, *PLCB1*, *SLC25A22, TBC1D24*] have been reported to cause EIMFS^2^,^3^,^4^,^5^,^6^,^7^, but the genetic causes of EIMFS are not fully elucidated.

The potassium (K^+^) -chloride (Cl^−^) co-transporter KCC2 encoded by *SLC12A5* (MIM ^*^606726) maintains low intracellular Cl^−^ concentrations ([Cl^−^]_i_) in neurons, and is essential for postsynaptic inhibition *via* activation of GABA_A_ and glycine receptors that are responsible for the Cl^−^ influx^8^. The presence of alternative first exons with different promoters provides two isoforms of KCC2a and KCC2b (see Fig. 1B). Mice deficient for both KCC2 isoforms die at birth due to severe motor defects, and KCC2b-specific knockout mice survive for up to 2 weeks, but die due to spontaneous seizures^9^,^10^,^11^, suggesting indispensable roles for KCC2 in proper mammalian brain function.

Recently, heterozygous missense mutations in *SLC12A5* were shown to be associated with febrile seizures and idiopathic generalized epilepsy in humans^12^,^13^, and very recently, autosomal recessive *SLC12A5* mutations were reported to cause EIMFS^14^. However, in the former two reports, the mutations were identified based only on the targeted DNA sequencing of *SLC12A5*, and possible causative mutations in other genes were not clearly excluded. In the more recent study^14^, all nonsynonymous mutations in the patients were systematically listed by whole exome sequencing (WES) analysis, and the *SLC12A5* mutations were selected as the most plausible causes based on several criteria. Nevertheless, the Cl^−^ extrusion function of KCC2 was not properly assessed in that study, as discussed in detail below. Therefore, the data did not allow for an estimation of intraneuronal Cl^−^ levels in the patients.

In this study, we identified novel compound heterozygous *SLC12A5* mutations in three families, including four affected individuals. Functional analysis using the gramicidin-perforated patch-clamp technique confirmed significant, but not complete, loss of KCC2 function in the patients. Individual mutations in each patient were found to impair KCC2 function to different degrees. Thus, our data demonstrated that partial loss of neuronal KCC2 function by biallelic mutations might cause migrating focal seizures, which are characteristic of EIMFS.

## Results

### Identification of biallelic *SLC12A5* mutations in individuals with EIMFS

**Figure 1:**
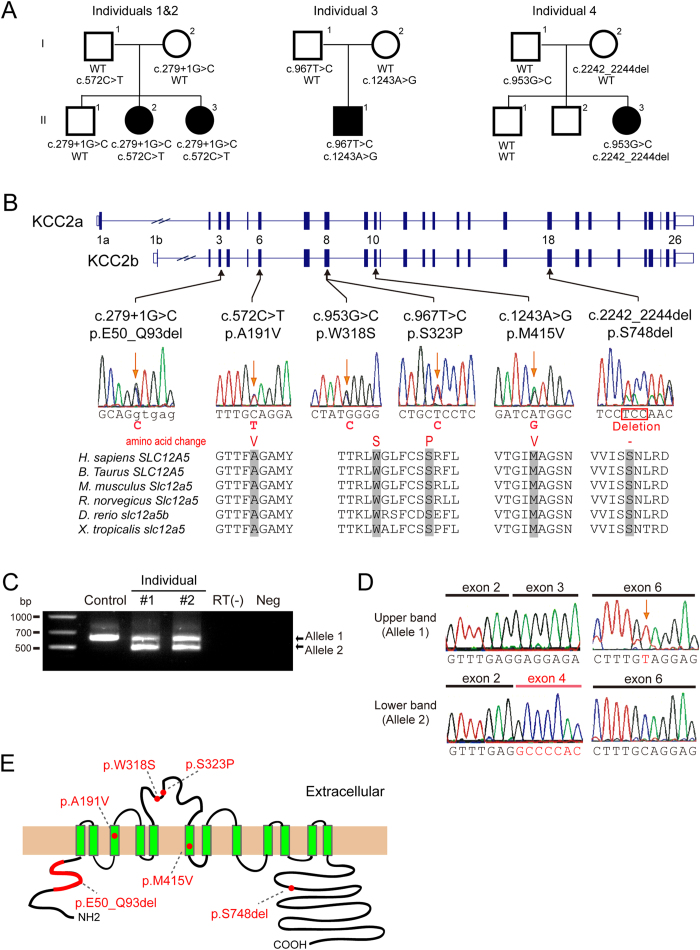
Biallelic *SLC12A5* mutations. (**A**) Familial pedigrees of four individuals with *SLC12A5* mutations. The segregation of each mutation is shown. (**B**) Schematic representation of *SLC12A5* (open and filled rectangles represent untranslated regions and coding regions, respectively) and its mutations. There are two transcriptional variants: variant 1 (GenBank accession number, NM_001134771.1) encoding KCC2a, variant 2 (NM_020708.4) encoding KCC2b. All missense mutations and an amino acid deletion (p.S748del) occur at evolutionarily conserved amino acids. Homologous sequences were aligned by the CLUSTALW website. (**C**) Reverse transcriptase-PCR analysis of individuals 1 and 2, and a control. Two PCR products representing transcripts from two alleles were detected in the individual cDNA, but only a single amplicon was detected in the control. (**D**) Sequence of upper (allele 1) and lower (allele 2) amplicons clearly show a c.572C > T mutation at exon 6 in allele 1 and deletion of exon 3 in allele 2. (**E**) Schematic presentation of the KCC2 protein^39^. Localization of the six mutations (red circle and bold lines) is shown.

To identify the genetic basis of autosomal recessive EIMFS, WES was performed in two Japanese siblings with EIMFS (individuals 1 and 2, Fig. 1A). A total of 309 and 272 rare protein-altering and splicing-affecting variants were identified per individual, in which 122 variants were common in two (Supplementary Table S1). We focused on genes with two heterozygous variants (possible compound heterozygous variants) or homozygous variants that were consistent with an autosomal-recessive trait, and found that *SLC12A5* was a solo candidate. Sanger sequencing validated the c.279 + 1G > C and c.572C > T (p.A191V) variants in two siblings, which were transmitted from their mother and father, respectively (Fig. 1A). The unaffected older brother had only the c.279 + 1G > C variant. We then searched the WES data of 10 sporadic cases with EIMFS for *SLC12A5* mutations, and found another Malaysian patient (individual 3) with compound heterozygous *SLC12A5* mutations: c.967T > C (p.S323P) and c.1243A > G (p.M415V) (Fig. 1A).

To investigate the possible involvement of *SLC12A5* mutations in other types of infantile epilepsy, we also searched the WES data of 526 patients for biallelic *SLC12A5* mutations, and examined an additional 141 patients by *SLC12A5-*targeted resequencing as a second cohort. Following *SLC12A5* resequencing, in which the mean depth of *SLC12A5* coding sequences was 244 (range 41 to 465), we identified a Japanese patient with compound heterozygous *SLC12A5* mutations [c.953G > C (p.W318S) and c.2242_2244del (p.S748del)], who was diagnosed as unclassified intractable epilepsy (individual 4, Fig. 1A). Other biallelic mutations were unidentified in the WES data of 526 epileptic patients. These six mutations were absent in dbSNP 138, our in-house 575 control exomes, the Exome Variant Server, and EXaC database (Supplementary Table S2). Four missense mutations and an in-frame amino acid deletion occurred at evolutionarily conserved amino acids (Fig. 1B). At least two of three web-based prediction tools (SIFT, Polyphen-2, and MutationTaster) predicted that the four missense mutations could affect protein function (Supplementary Table S2).

To examine the mutational effect of c.279 + 1G > C, reverse transcriptase-PCR was performed using total RNA from lymphoblastoid cell lines (LCLs) derived from individuals 1 and 2. Results demonstrated that the c.279 + 1G > C mutation caused a deletion of exon 3 from the *SLC12A5* mRNA, resulting in an in-frame 44-amino acid deletion (p.E50_Q93del) (Fig. 1C,D). All six mutations were located on both KCC2a and KCC2b (Fig. 1B,E), and affected the N− and C− terminal regulatory domains (p.E50_Q93del and p.S748del, respectively)^15^, transmembrane domains (p.A191V and p.M415V), and the large extracellular loop (p.W318S and p.S323P) adjacent to four conserved cysteines (C287, C302, C322, C331), which is required for KCC2 activity^16^.

### Differentially impaired Cl^−^ extrusion function of two KCC2 mutants in individual epileptic patients

**Figure 2:**
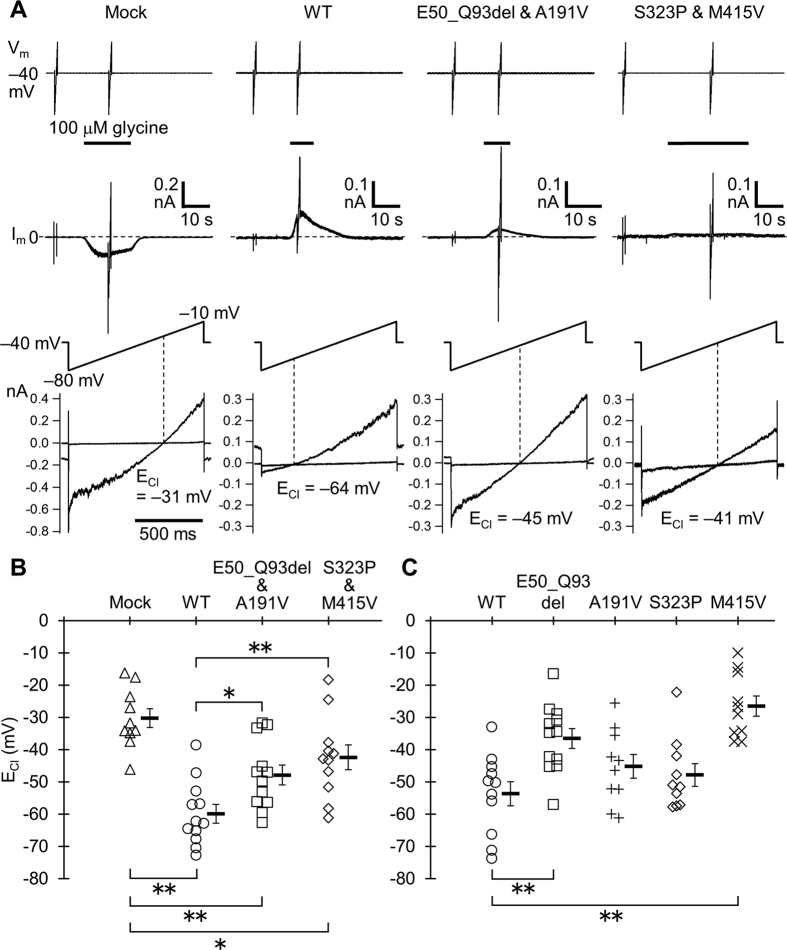
E_Cl_ in WT and mutant KCC2-expressing HEK293-GlyRα1 cells. (**A**) Representative traces of GlyR currents in cells co-transfected with two different vectors encoding only EGFP and DsRed (Mock), WT-KCC2 (WT), two KCC2 mutants expressed in individuals 1 and 2 (E50_Q93del & A191V), and mutants in individual 3 (S323P & M415V). Currents were recorded under the gramicidin-perforated voltage-clamp condition. Upper traces indicate membrane voltage (V_m_) changes. The holding voltage was −40 mV. Two 1-s voltage ramps from −80 to −10 mV were applied before and during bath application of 100 μM glycine. Middle traces show membrane current (I_m_) responses. The humps of GlyR currents were generated during glycine application at the holding voltage of −40 mV, and the current responses to voltage ramps were generated before and during the humps. Note that the current levels immediately before and after a ramp response during a GlyR current hump were almost unchanged, and therefore the time course of the humps was not affected by ramp responses. This confirmed that the net Cl^−^ flux across the cell membrane during a ramp response did not significantly alter E_Cl_. See also Supplementary Fig. S1. Bottom traces are the expanded traces of single voltage ramps (upper traces) and superimposed current responses to voltage ramps before and during glycine application (lower traces). Dotted lines indicate the voltage levels at which the superimposed current traces intersected, corresponding to E_Cl_. (**B**) Plot of E_Cl_ in cell groups of Mock (*n* = 10), WT (*n* = 12), E50_Q93del & A191V (*n* = 12), and S323P & M415V (*n* = 11). *P < 0.03, **P < 0.01 by REGW *F*-test. (**C**) Plot of E_Cl_ in cells transfected with single vectors encoding WT (*n* = 11), E50_Q93del (*n* = 12), A191V (*n* = 10), S323P (*n* = 10), and M415V (*n* = 10). **P < 0.01 by Dunnett’s two-sided *t*-test.

To assess the mutational effects of KCC2 on Cl^−^ extrusion function, the HEK293 cells stably expressing the α1 type glycine receptor (GlyR)^17^ were transfected with the mutants or wildtype (WT) KCC2. Then we compared reversal potentials of GlyR-mediated Cl^−^ currents, which reflect the equilibrium potentials for Cl^−^ (E_Cl_), *i.e.* [Cl^−^]_i_ controlled by KCC2, in the transfected cells using the gramicidin-perforated patch-clamp technique. We used a voltage ramp from −80 to −10 mV and determined E_Cl_ as the voltage level at which the GlyR current became zero, corresponding to the level at the intersection of superimposed current traces obtained before and during application of 100–300 μM glycine (Fig. 2A and Supplementary Fig. S1; inward and outward currents beyond the intersection indicate efflux and influx of Cl^−^ through GlyRs, respectively. See Materials and Methods and the legend of Supplementary Fig. S1 for details). Thus, a greater negative E_Cl_ indicated greater extrusion of Cl^−^ by KCC2.

First, we co-transfected the cells with a pair of two different KCC2 mutants, *i.e.* a pair of E50_Q93del and A191V mutants or a pair of S323P and M415V mutants, mimicking the condition in individuals 1 and 2 or individual 3, respectively. We confirmed that the E_Cl_s in cells expressing the two mutants in individuals 1 and 2 (−47.9 ± 3.1 mV, *n* = 12) and in individual 3 (−42.3 ± 3.9 mV, *n* = 11) were significantly more positive than the E_Cl_ in WT-expressing cells (−59.9 ± 2.9 mV, *n* = 12; Fig. 2B). However, the E_Cl_s in mutant-expressing cells were significantly more negative than in cells transfected with the vector containing no KCC2 (Mock, −30.2 ± 2.9 mV, *n* = 10; Fig. 2B). Thus, the Cl^−^ extrusion function of KCC2 was partially reduced by mutations in cells mimicking patient status.

To determine whether individual KCC2 mutants contributed equally to the reduction, we also measured and compared E_Cl_s in cells only transfected with one type of KCC2 mutant. We found that the E_Cl_s in cells expressing the E50_Q93del mutant in individuals 1 and 2 (−36.5 ± 3.1 mV, *n* = 12) and in the cells expressing the M415V mutant in individual 3 (−26.5 ± 3.2 mV, *n* = 10) were much more positive than that in WT-expressing cells (−53.6 ± 3.8 mV, *n* = 11). The E_Cl_s in cells expressing the A191V mutant in individuals 1 and 2 (−45.2 ± 3.7 mV, *n* = 10) and in the cells expressing the S323P mutant in individual 3 (−47.8 ± 3.5 mV, *n* = 10) also seemed to be more positive than that in WT-expresing cells, but did not reach statistical significance (Fig. 2C). Thus, individual patients were found to have one KCC2 mutant with a severely impaired Cl^−^ extrusion function and a second mutant with mildly impaired Cl^−^ extrusion function.

### Unaltered cellular distribution and cell surface expression of KCC2 by the mutations

**Figure 3:**
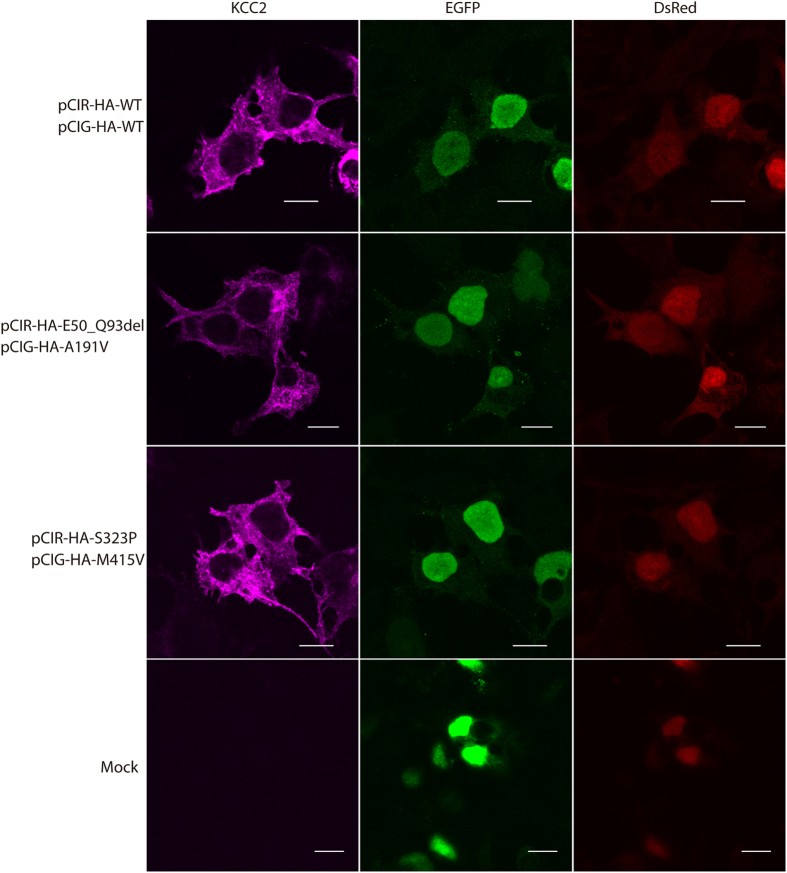
Cellular distribution of KCC2 mutants in transfected HEK293 cells. Confocal immunofluorescence images of KCC2 in HEK293 cells co-expressing pCIR-HA-WT and pCIG-HA-WT (uppermost row), pCIR-HA-E50_Q93del and pCIG-HA-A191V (2nd row), pCIR-HA-S328P and pCIG-HA-M415V (3rd row), and only pCIG-HA and pCIR-HA (Mock; lowermost row). Cotransfection of HEK293 cells was confirmed by the presence of EGFP (green) and DsRed (red) in the nucleus. Similar expression patterns of KCC2 (pink) were observed in WT- and mutant-expressing cells. KCC2 immunofluorescence was not observed in mock-transfected cells. Scale bars represent 10 μm.

To address the cause of reduced Cl^−^ extrusion function of KCC2 mutants, we next compared the cellular distribution of KCC2 mutants with WT KCC2 using immunofluorescence staining. In all WT-expressing and mutant-expressing HEK293 cells mimicking conditions in normal control, individuals 1 and 2 (E50_Q93del and A191V), and individual 3 (S323P and M415V), and also in the cells expressing individual mutants, KCC2 immunoreactivity was similarly detected in the plasma membrane and the perinuclear region (Fig. 3 and Supplementary Fig. S2). This suggests that the mutations did not greatly alter KCC2 subcellular localization.

**Figure 4:**
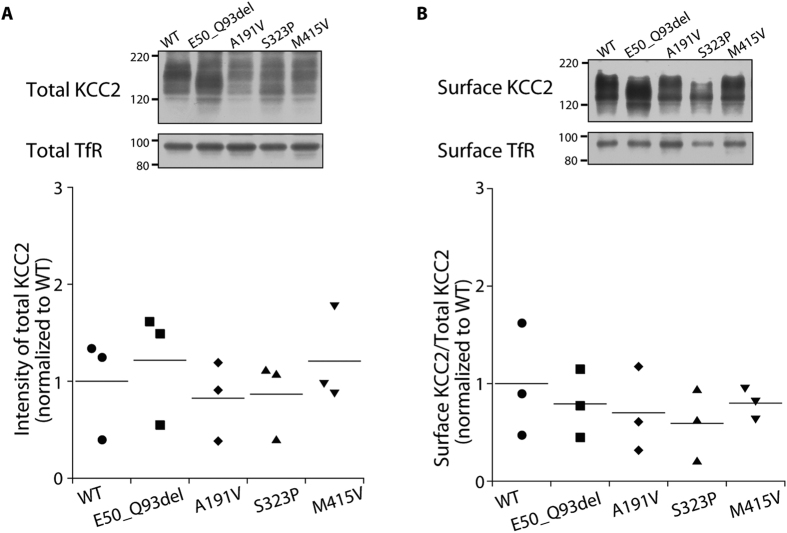
Cell surface and total expression levels of KCC2 mutants, as measured by the surface biotinylation and immunoblotting assay of KCC2 and transferrin receptor (TfR). (**A**) Upper panels show representative immunoblots of total KCC2 and total TfR. In the dot plot, the total KCC2 levels were normalized to total TfR levels in each type of transfected cell. There were no significant differences in mean total KCC2 level between WT- and mutant-expressing cells (*P* = 0.7835, *n* = 3). (**B**) Upper panels show representative immunoblots of surface KCC2 and surface TfR. The dot plot shows the ratios of surface KCC2 levels to total KCC2 levels in each type of transfected cell, which were further normalized to the mean ratio in WT-expressing cells. There are no significant differences in the normalized ratio between WT- and mutant-expressing cells (*P* = 0.7899, *n* = 3).

We also compared total and cell surface expression levels of individual KCC2 mutants with those of WT KCC2 using the surface protein biotinylation and immunoblotting assay. Results showed no clear differences in total KCC2 expression between WT- and mutant-expressing cells (Fig. 4A). Moreover, the differences in the ratio of surface expression to total KCC2 expression between WT- and mutant-expressing cells did not reach statistical significance (Fig. 4B). Therefore, mutations in the patients did not greatly alter total or cell surface expression levels of KCC2.

### Clinical features

**Figure 5:**
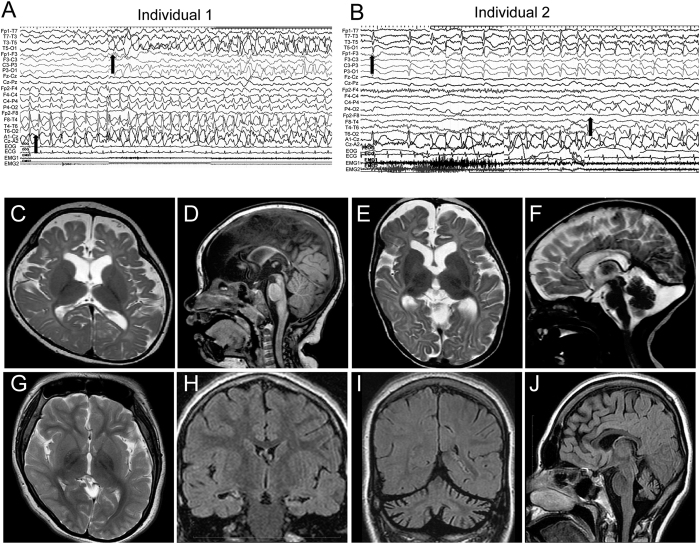
Clinical features of individuals with biallelic *SLC12A5* mutations. (**A**) Ictal EEG of individual 1. The initial spikes over the right frontal area (lower arrow) were accompanied by eye deviation to the left, then the left temporal spikes emerged (upper arrow) with subsidence of the right frontal spikes, which were accompanied by eye deviation to the right. (**B**) Ictal EEG of individual 2. The initial spikes over the left temporal area (upper arrow) were accompanied by a tonic seizure of the right upper extremity, then the right parietal spikes emerged (lower arrow) with subsidence of the left temporal spikes, which was accompanied by a tonic seizure of the left upper extremity. (**C–J**) Brain MRI of individual 1 at 13 months of age (**C,D**), individual 2 at 5 months (**E,F**), and individual 4 at 20 years of age (**G–J**). T2-weighted images (**C,E–G**) and T1-weighted images (**D,J**) and fluid-attenuated inversion recovery images (FLAIR) (H and I) are shown. Thin corpus callosum, frontal and temporal lobe atrophy, and delayed myelination were commonly observed in individuals 1 and 2 (**C–F**). Arachnoid cyst in the left posterior fossa was observed in individual 2 (**F**). Delayed myelination in the subcortical white matter of the temporal lobe was observed in individual 4 (**G**). Inferior horns of the lateral ventricle were mildly dilated and bilateral hippocampi were hypoplastic with slightly high signal intensity on FLAIR coronal view (**H**), indicating hippocampal sclerosis. Atrophic change of the cerebellar hemisphere (**I**) and vermis (**J**) was evident.

Clinical features of four individuals with biallelic *SLC12A5* mutations are summarized in Table 1, and case reports are available in the supplementary material. The onset of seizures was within 2 months of age, and various types of focal seizures were observed. Ictal electroencephalography (EEG) showed alternating seizures, accompanied by migrating foci from one hemisphere to the other, in three individuals with EIFMS (Fig. 5A,B). Interictal EEG was initially normal in 2 of 3 patients, and subsequently showed slow-wave activity or focal epileptic discharges compatible with EIFMS. Individual 4 was not definitively diagnosed with EIMFS, due to the lack of former medical records including ictal electroencephalograms. However, multifocal seizures, which started at variable parts of the body and migrated to other regions, were observed during infancy by her parents, which strongly suggested EIFMS. All four individuals exhibited severe developmental delay, hypotonia, and postnatal microcephaly. Brain MRI showed cerebral atrophy in four individuals, and delayed myelination and thin corpus callosum in three and two individuals, respectively. Individual 4 showed progressive cerebellar atrophy and hippocampal atrophy (Fig. 5C–J). The combination of potassium bromide and high-dose phenobarbital was effective for individuals 1 and 2, and a ketogenic diet controlled seizures in individual 3. Seizures of individual 4 were intractable.

## Discussion

In this study, we identified four patients exhibiting severe infantile epileptic seizures with compound heterozygous mutations in *SLC12A5*, which encodes the neuronal K^+^-Cl^−^ co-transporter KCC2. Three of the patients were diagnosed with EIMFS, and multifocal migrating seizures were also observed in an additional patient (individual 4). Together with the previous report^14^, our data strongly indicate that biallelic *SLC12A5* mutations cause migrating focal seizures, which is characteristic of EIMFS.

Functional analysis of the four KCC2 mutants revealed that each of the three patients (individuals 1–3) has two KCC2 mutants exhibiting differentially impaired Cl^−^ extrusion function. The combination of mutants caused an average positive E_Cl_ shift by 12–18 mV from the E_Cl_ in WT-expressing cells (Fig. 2B), corresponding to an increase in [Cl^−^]_i_ by 10–15 mM. This positive E_Cl_ shift in neuronal populations could result in an increased fraction of neurons that exhibit an excitatory response to GABA^18^,^19^,^20^,^21^. In the brains of patients with temporal lobe epilepsy, interictal discharge events detected during local field recording or intracranial EEG recording in epileptic foci are preceded by synchronous burst firing of GABAergic interneurons, and the firing triggers not only inhibitory postsynaptic potentials in most pyramidal neurons but also out-of-phase firing of a proportion (~20%) of pyramidal neurons exhibiting depolarizing GABA responses^19^,^20^,^21^,^22^. This out-of-phase firing contributes to the pathological high-frequency fast ripple component of the discharges^23^,^24^. Thus, similar out-of-phase firing activities might also take place in the brains of our patients, which would lead to a migrating focal seizure event when additional vulnerability factors are imposed on a part of the brain^20^,^21^,^22^,^23^,^24^. A significant suppression of seizures with a high dose of phenobarbital, a GABA_A_ receptor enhancer, in our patients (individuals 1 and 2; but its effect was temporary; see Table 1 and Supplementary Information for details) implies that strong enhancement of inhibitory GABA action on surrounding neurons is necessary for diminishing the effect of out-of-phase firing, even of a minor proportion of neurons.

Individual mutations found in this study did not greatly alter cellular distribution (Fig. 3 and Supplementary Fig. S2), surface or total expression levels of KCC2 (Fig. 4). Nevertheless, our immunostaining and blotting would have not resolved subtle differences in the surface expression level, which might explain the subtle differences in Cl^−^ extrusion function especially between p.A191V and p.S323P mutants and WT KCC2. The surface expression of KCC2 may be determined by the phosphorylation/dephosphorylation balance of the residues, especially in the cytoplasmic C-terminal domain of KCC2^15^. The locations of the mutations examined in this study, which were the N-terminal domain (p.E50_Q93del), transmembrane domains (p.A191V and p.M415V) and the large extracellular loop (p.S323P), thus might have less effect on trafficking of KCC2 to the surface, although the effect of p.S748del located in the C-terminal domain in individual 4 has not yet been examined. It is also possible that the impaired Cl^−^ extrusion function of our mutants would be due to impairment in an intrinsic transport property of KCC2. Although the tertiary structure and ion-transporting structural element of KCC2 remain unknown, the mutations may affect Cl^−^ binding of KCC2 or cause some derangement in the KCC2 structure, thereby impairing Cl^−^ extrusion. Further studies are needed to elucidate whether individual mutations affect either or both the trafficking and the intrinsic transport property of KCC2.

The KCC family members are thought to function as oligomers^15^. A previous report about a KCC3 mutant defective in its surface expression suggested its dominant-negative effect on the expression of not only WT-KCC3 but also WT-KCC2 through forming heteromers^25^. Thus it might be possible that individual KCC2 mutants found in this study exert some effect on other mutants or WT-KCC2 through oligomerization. We have not determined to what extent such interactions actually work in patients and also in heterozygous healthy parents. However, given that the mean E_Cl_s in the cells expressing KCC2 mutants as in the patients (Fig. 2B) were similar to or more negative than the average of the mean E_Cl_s in cells expressing individual mutants (Fig. 2C), the severely impaired mutants of E50_Q93del and M415V would not have a strong dominant-negative effect. This further implies that the mean neuronal [Cl^−^]_i_ level in heterozygous healthy parents would be lower than that in patients, because of the presence of normal KCC2 in the parents. However, the differences in neuronal [Cl^−^]_i_ between the parents and the patients or normal controls, if any, would be too small to detect by our E_Cl_ measurement. In any case, our genetic evidence certainly indicated that compound heterozygous mutations in *SLC12A5* only match the presence of symptoms in the patients. Thus, even though the mean neuronal [Cl^−^]_i_ level in the heterozygous parents might actually be slightly higher than that in normal controls, it should not be the level causing diseases. Therefore, the number of neurons firing out-of-phase, which predispose an individual to an ictal event, would not be significantly increased in the parents.

A recent study also reported four infantile patients exhibiting migrating focal seizures with mutations in *SLC12A5*^14^. Two patients had compound heterozygous missense mutations c.1277T > C (p.L426P) and c.1652G > A (p.G551D), and the other two (one deceased) possessed a homozygous missense mutation c.932T > A (p.L311H). Functional analysis concluded that the Cl^–^ extrusion function of L426P and G551D mutants was completely lost, whereas the L311H mutant was still partly functional, and that these functional losses were due to reduced surface expression and glycosylation of these mutants^14^. However, the study measured E_Cl_ under whole-cell patch-clamp conditions, in which the basal [Cl^−^]_i_ during recordings was determined by the Cl^−^ concentration in the pipette solution. Therefore, the data did not provide information about the impact of the mutations on neuronal [Cl^−^]_i_ levels in the patients. Moreover, the authors used whole-cell pipette solution containing 110 mM Cs^+^, instead of K^+^. KCC2 excludes Cl^−^ with K^+^ out of the cells using the K^+^ transmembrane gradient, but the replacement of intracellular K^+^ with Cs^+^ is known to block KCC2-mediated Cl^−^ extrusion^26^,^27^,^28^. Therefore, in the preceding study^14^, KCC2 activity must have been inhibited and E_Cl_ would have not been correctly recorded. Conversely, using Cl^−^-impermeable gramicidin channels as the current mediator incorporated into the patch membrane, our study clearly demonstrated, for the first time, that E_Cl_ in cells expressing the KCC2 mutants in patients shows a positive shift, but remains more negative than expected E_Cl_ level in the absence of KCC2 (Fig. 2B). Therefore, we confirmed that KCC2 mutant function in our patients was reduced, but still functional, although the collectively reduced function of two mutant alleles is sufficient to cause severe epileptic seizures. The onset of seizures in patients within a few days after birth (Table 1) was compatible with the period of increasing functional KCC2 expression^29^,^30^,^31^,^32^,^33^,^34^, and this may also support our conclusion.

In conclusion, our data demonstrated that individual mutations in EIMFS patients causes variable loss of KCC2 function, and that the combinatory effect of partial loss of KCC2 function in each patient results in focal seizures, severe developmental delays, and postnatal microcephaly.

## Materials and Methods

### Patients

A total of 10 sporadic cases and one family with two affected siblings, who had an initial diagnosis of EIMFS, were analyzed by WES as an initial cohort. Patients with mutations in known epilepsy genes related to EIMFS (*KCNT1*, *SCN1A*, *SCN2A*, *SCN8A*, *PLCB1*, *SLC25A22,* and *TBC1D24*)^2^,^3^,^4^,^5^,^6^,^7^ were excluded from the study. Additionally, we searched WES data from 526 patients with infantile epilepsy, and examined 141 patients with infantile epilepsy by *SLC12A5-*targeted resequencing as a second cohort. DNA was extracted from peripheral blood leukocytes using standard methods. DNA was extracted from saliva samples from the father and elder brother of individuals 1 and 2, as well as from the elder brother of individual 4, using Oragene (DNA Genotek). Detailed clinical information was obtained from corresponding clinicians. Written informed consent was obtained for all individuals. Experimental protocols were approved by the Institutional Review Board of Yokohama City University School of Medicine, and were carried out in accordance with the approved guidelines.

### Genetic analysis

Genomic DNA was captured using the SureSelect Human All Exon v5 Kit (Agilent Technologies), and sequenced on HiSeq2500 (Illumina) with 101 bp paired-end reads. Exome data processing was performed as previously described^35^. To identify novel genetic causes for EIMFS, we focused on rare nonsynonymous variants with minor allele frequencies below 1% in dbSNP135 data, and variants were not found in more than five of our in-house 575 control exomes. For *SLC12A5* resequencing, due to the insufficient amount of genomic DNA, whole genomic amplification using the Illumina GenomiPhi V2 DNA Amplification Kit (GE Healthcare Japan, Tokyo, Japan) was performed. *SLC12A5* coding exons were amplified by PCR using KOD FX Neo DNA polymerase (Toyobo), with amplified DNA as the template. DNA libraries were prepared by using the Nextera DNA Sample Preparation Kit (Illumina) and sequenced on the MiSeq (Illumina) with 150 bp paired-end reads. *SLC12A5* variants were annotated based on transcript variant 2 (encoding KCC2b, NM_020708.4), and were validated by Sanger sequencing using genomic DNA.

### Reverse transcriptase-PCR

LCLs were established from individuals 1 and 2. Total RNA was extracted using the RNeasy Plus Mini kit (Qiagen) from LCLs. A total of 4 μg total RNA was subjected to reverse transcription, and 2 μl cDNA was used for PCR. PCR conditions and primer sequences are shown in Supplementary Table S3. PCR products were electrophoresed on a 1.5% agarose gel. PCR bands were cut from the gel, purified using the QIAEXII Gel Extraction Kit (Qiagen), and sequenced.

### Expression vectors

A full-length human cDNA of *SLC12A5* transcript variant 2 (clone ID: RC223680) was obtained from Origen (Rockville, MD). Site-directed mutagenesis using a KOD-Plus-Mutagenesis kit (Toyobo) was used to generate *SLC12A5* mutants, including c.148_279del (p.E50_Q93del), c.572C > T (p.A191V), c.967T > C (p.S323P) and c.1243A > G (p.M415V). All variant cDNAs were verified by sequencing. WT and mutant *SLC12A5* cDNAs were cloned into either the pCIG-HA or pCIR-HA vector, in which a N-terminal HA-tag sequence was introduced by PCR to parental pCIG or pCIR vectors^36^,^37^ to express N-terminal HA-tagged KCC2b as well as nuclear-localized EGFP or DsRed. Co-expression of different mutants was confirmed by the presence of both EGFP and DsRed in the nucleus.

### Cell culture and transfection

A stable HEK293 cell line expressing GlyRα1 (HEK293-GlyRα1) was generated as previously described^17^, except for the use of the pCMV-GlyRα1WT vector^38^. The cells were maintained in Dulbecco’s minimum essential medium (Sigma) supplemented with 10% fetal bovine serum, 100 units/mL penicillin, 100 μg/mL streptomycin, and 400 μg/ml G418. For single- or double-transfection of cells with the indicated cDNA, lipofectamine 3000 (Invitrogen) was used according to the manufacture’s protocol. Cells were used 2–3 days after transfection.

### Electrophysiology

Membrane currents under the gramicidin-perforated voltage-clamp condition were recorded through an EPC10 amplifier controlled via Patchmaster software (HEKA Elektronik). Records were filtered at 1 kHz and digitized at 5 kHz. Patch pipettes were fabricated from borosilicate glass capillaries using a P-97 puller (Sutter Instrument). Pipette resistance was 2–4 MΩ when filled with the pipette solution containing (in mM): 145 KCl, 5 K-HEPES, 6 HEPES (pH 7.4, 280 mOsm/kg H_2_O), and 50 μg/ml gramicidin. The extracellular solution contained (in mM): 145 NaCl, 5 KCl, 2 CaCl_2_, 1 MgCl_2_, 5 Na-HEPES, 6 HEPES (pH 7.4, 300 mOsm/kg H_2_O), and 10 μM bumetanide to block endogenous Na^+^ -K^+^ -2Cl^−^ cotransporters in HEK293 cells. The liquid junction potential between these solutions was 2.8 mV and was corrected online. Cells were placed on a small glass-bottom recording chamber filled with 0.5 ml of external solution, and the cells expressing nuclear EGFP and/or DsRed were selected under epifluorescent illumination. A > 5 GΩ (usu. ∼10 GΩ) gigaseal was first formed, and then we typically waited for 1–1.5 hours until the series resistance (Rs) was reduced to <100 MΩ by insertion of gramicidin into the patch membrane before recordings. The Rs during recordings was compensated for by 70%. Our high-quality gigaseal recording resulted in a very low basal current level (6.8 ± 0.8 pA in absolute value, *n* = 98) at E_Cl_. Thus IR-drop errors through Rs (81.5 ± 4.3 MΩ) with 70% Rs compensation were 0.16 ± 0.02 mV in absolute value, indicating negligible IR-drop errors in our E_Cl_ values. The holding voltage was set at −40 mV and 1 s voltage ramps from −80 to −10 mV were applied before and during bath application of 100–300 μM glycine. The current levels immediately before and after a voltage ramp during a glycine-induced current hump were almost unchanged (Fig. 2A and Supplementary Fig. S1), confirming that the net Cl^−^ flux across the cell membrane during a voltage ramp did not significantly alter E_Cl_. E_Cl_ measurements in a single cell were repeated more than 3 times at >3 min intervals (Supplementary Fig. S1), and the average over three successive measurements was adopted as the final E_Cl_ value. This value was plotted in the graphs in Fig. 2B,C. When the variation of three successive E_Cl_ values did not converge within ±1 mV, the cell was discarded from the data. All experiments were performed at 26–28 °C.

### Immunofluorescence staining

WT KCC2 and KCC2 mutants were transiently expressed in HEK293-GlyRα1 cells, fixed with 4% paraformaldehyde in PBS, permeabilized with 0.3% Triton X-100, and then blocked with 2% bovine serum albumin. The cells were then incubated overnight at 4 °C with primary antibodies specific to KCC2 (1:325, Millipore, #07–432) and GFP (1:500, Aves labs), and an anti-RFP antibody that also recognized DsRed (MBL, 1:100). The fluorescent Alexa Fluor-conjugated secondary antibody (1:300, Invitrogen) was then applied for 2 h at room temperature. Coverslips were mounted in PermaFluor aqueous mounting medium (Thermo Scientific), and the immunofluorescent images were acquired with a confocal laser-scanning microscope (FV1000-D, Olympus).

### Immunoblotting

Surface biotinylation experiments were performed using a Pierce Cell Surface Protein Isolation kit (Thermo Fisher Scientific) according to the manufacturer’s protocol. Briefly, HEK293 cells expressing WT KCC2 or KCC2 mutants were washed with ice-cold PBS and then labeled with 0.25 mg/ml sulfo-NHS-SS-biotin for 30 min at 4 °C. Excess biotin was quenched with glycine buffer. The cell lysates were centrifuged (10,000 *g* for 10 min), the supernatant was isolated with NeutrAvidin gel, and the bound proteins were then eluted with SDS-PAGE sample buffer. Total cell lysate and biotinylated proteins were separated by SDS-PAGE and transferred to a nitrocellulose membrane. The blots were blocked in 1% bovine serum albumin and incubated overnight at 4 °C with following primary antibodies: rabbit anti-KCC2 (1:1000, Millipore, #07–432), and mouse anti-transferrin receptor (TfR) (1:500, clone H68.4, Zymed Laboratories). The cells were then incubated with horseradish peroxidase-conjugated secondary antibody (GE Healthcare) for 1 h at room temperature. Immunoblots were visualized with enhanced chemiluminescence (ECL) exposed onto Polaroid instant films through the ECL Mini-camera (GE Healthcare). Band intensities were measured using ImageJ software. Surface and total KCC2 band densities were normalized to the TfR band density. TfR is a membrane protein unrelated to KCC2 and was used as a loading control.

### Statistics

Statistical analyses of E_Cl_ data were assessed using IBM SPSS ver.21 software. The Kolmogorov-Smirnov test and the Levene statistic confirmed the normality of data distribution and homogeneity of variances, respectively, for all data in Fig. 2B,C. Multiple comparisons were made using one-way ANOVA followed *post-hoc* by Ryan–Einot–Gabriel–Welsch (REGW) *F*-test in Fig. 2B and by Dunnett’s two-sided *t*-test in Fig. 2C. The multiple comparisons in Fig. 4 were made using one-way ANOVA. Data are presented as mean ± SEM.

## Additional Information

**How to cite this article**: Saitsu, H. *et al*. Impaired neuronal KCC2 function by biallelic *SLC12A5* mutations in migrating focal seizures and severe developmental delay. *Sci. Rep.* **6**, 30072; doi: 10.1038/srep30072 (2016).

## Supplementary Material

Supplementary Information

## Figures and Tables

**Table 1:** Clinical features of individuals with *SLC12A5* mutations.

	Individual 1 (Japanese sib1)	Individual 2 (Japanese sib2)	Individual 3 (Malaysian)	Individual 4 (Japanese)
Age, gender	4 years 8 months, female	3 years 1 month, female	1 year 11 months, male	20 years, female
Mutations	c.279 + 1G > C, p.Glu50_Gln93del; c.572C > T, p.Ala191Val	c.279 + 1G > C, p.Glu50_Gln93del; c.572C > T, p.Ala191Val	c.967T > C, p.Ser323Pro; c.1243A > G, p.Met415Val	c.953G > C, p.Trp318Ser; c.2242_2244del, p.Ser748del
Diagnosis	EIFMS	EIFMS	EIFMS	Intractable epilepsy (Possible EIFMS)
Initial symptom	Clonic seizure followed by tonic phase at day 1	Tonic seizure at day 3	Apneic episodes at 1.5 months	Upward eye deviation and cyanosis at day 1
Initial interictal EEG	Normal sleep background activity with slow waves over the left posterior area at 2 months	Normal sleep background activity at 2 months	Normal at 2 months	Unknown
Course of seizures	Focal tonic seizures at day 6; apnea, asymmetrical tonic posture with flushed face, twitching of fingers, left or right eyelid or mouth, eye deviation to the left or right at 1 month; seizure-free between 16 and 40 months; tonic seizures with vocalization, motion arrest with staring since 3 years 4 months	Upward eye deviation, tonic posture of arms followed by pedaling movements at day 3; apnea and clonic seizures of the left or right extremities at 1 month; seizure-free between 3 to 11 months; clonic seizures of one extremity evolving into other extremities, hyperventilation, tonic extension of the arms with apnea since 11 months	Apneic episode with loss of consciousness at 2 months; tonic seizures of extremities at 2.5 months; cyanotic starry-eyed episodes at 5 months; bilateral eye gazing and deviation of the head to either the right or the left, focal clonic movements involving different limbs at 6 months; brief, blank stare, deviation of eyes to one side, tonic posturing at 7 months	Clonic-tonic seizure at 2 days; upward eye deviation, tongue spasm, tonic posturing of unilateral extremities, tonic-clonic seizure at 6 months
Follow-up EEG	Runs of increment rhythmic θ activity over the bilateral frontocentroparietal areas during sleep at 13 months; occasional runs of HVS over the left frontal area and several spikes over the right frontal area during sleep at 3 years 6 months	Generalized high-voltage slow background of 2 Hz delta activity and no epileptiform activity during wakefulness at 1 year 9 months	Diffusely attenuated background with ventilation artifact at 6 months; normal EEG background with some spikes over the frontal region at 9 months; no electrographic seizures	Sharp waves over the left central and frontal region at 10 years; slow back ground activity of 5 Hz
Effective drugs	KBr, high-dose PB, AZM for apnea	KBr, high-dose PB, VPA	TPM, LEV, KD	Intractable (none)
Head control	1 year, but still unstable at 4 years 8 months	—	4 months to 5.5 months, regressed and lost head control from 5.5 months, regained some head control by 11 months.	1 year
Rolling over	2 years 6 months, but incomplete at 4 years 6 months	—	4 months to 5.5 months, regressed after 5.5 months, able to roll over again at 9–10 months	2 years
Sitting	+	—	Sits unsupported by 23 months, but still slightly unsteady	—
Meaningful words	—	—	—	—
Muscle tonus	Hypotonia	Hypotonia	Hypotonia	Hypotonia
Involuntary movements	—	—	—	—
Head circumference	34.0 cm (+0.3 SD) at birth; 43 cm (−4.7 SD) at 4 years 8 months	33.7 cm (+0.7 SD) at birth; 45.2 cm (−2.0 SD) at 3 years	35 cm (25^th^ centile) at birth; 46cm (3^rd^ centile) at 23 months	33 cm (−0.1 SD) at birth; 47.2 cm (−4.0 SD) at 10 years
Brain MRI	Thin corpus callosum, frontal and temporal lobes atrophy, delayed myelination at 2 months; same findings at 13 months	Thin corpus callosum, frontal lobe atrophy, delayed myelination, arachnoid cyst in the left posterior fossa at 5 months	Mild brain atrophy at 3 months	Subdural hygroma; bilateral hippocampal atrophy with high intensity on FLAIR image, mild atrophy of the cerebellum, delayed myelination of the temporal lobe at 10 years; progression of cerebellar atrophy at 20 years

EEG, electroencephalography; TPM: topiramate; LEV, levetiracetam; KD, ketogenic diet; KBr, potassium bromide; PB, phenobarbital; AZM, acetazolamide; VPA, valproic acid; HVS, high-voltage slow waves.
