# Supplementary material for: Impaired neuronal KCC2 function by biallelic SLC12A5 mutations in migrating focal seizures and severe developmental delay
Source: Sci Rep. 2016 Jul 20;6:30072. doi: 10.1038/srep30072 (PMC4951812; doi:10.1038/srep30072)
Supplement: Supplementary Information [file srep30072-s1.doc]

**­Supplementary Information**

**Impaired neuronal KCC2 function by biallelic *SLC12A5* mutations in migrating focal seizures and severe developmental delay**

Hirotomo Saitsu, Miho Watanabe, Tenpei Akita, Chihiro Ohba, Kenji Sugai, Winnie Peitee Ong, Hideaki Shiraishi, Shota Yuasa, Hiroshi Matsumoto, Khoo Teik Beng, Shinji Saitoh, Satoko Miyatake, Mitsuko Nakashima, **Noriko Miyake,** Mitsuhiro Kato, Atsuo Fukuda and Naomichi Matsumoto

**Case reports**

*Individuals 1 & 2*

Individual 1 is a 4-year-old Japanese girl, who was born to non-consanguineous parents as a second child after an uneventful full-term pregnancy. Her elder brother showed afebrile convulsion once at 1 year of age. Her Apgar scores at 1 and 5 minutes were 8 and 9, respectively. The birth weight was 3,054 g (−0.5 standard deviation [SD]). Clonic seizures of the upper extremities, followed by tonic seizures, developed at the second day of life. Seizures were temporarily controlled by intravenous phenobarbital (PB), but tonic seizures relapsed at 6 days of age. Tonic seizures improved with increased oral PB, but she exhibited focal motor seizures characterized by twitching of the right hand, evolving to right arm, right leg, mouth, and the other side of the extremities, lasting several tens of seconds to several minutes. These seizures were uncontrolled with combination of PB, carbamazepine (CBZ), and clonazepam (CZP), and she was admitted to our hospital at 59 days of age. Multiple seizure symptoms were observed, including apnea, asymmetrical tonic posturing with flushed face, twitching of the fingers, left or right eyelid, or mouth, and eye deviation to the left or right. These seizures were clustered and shifted with each other. Video-electroencephalography (V-EEG) at 2 months of age demonstrated alternating seizures accompanied by migrating foci from one area to another (Fig. 5A), although interictal EEG showed normal sleep background activity with slow waves over the left posterior area. Seizures were controlled with a combination therapy of high-dose PB and potassium bromide (KBr), and apneic seizures were suppressed with acetazolamide (AZM). Brain magnetic resonance imaging (MRI) at 2 months revealed a thin corpus callosum, frontal and temporal lobe atrophy, and delayed myelination, which were unchanged in the follow-up MRI at 13 months (Fig. 5C, D). Her development was severely delayed; she gained visual pursuit and babbling at 6 and 11 months of age, respectively. Head control and rolling-over were obtained at 1 year and 2 years of age, respectively, but both were still unstable and incomplete even at the present age. Interictal EEG at 13 months showed runs of increment rhythmic θ activity over the bilateral frontocentroparietal areas during sleep. She experienced near-seizure freedom from 1 year 4 months, but tonic seizures with vocalization and motion arrest with staring relapsed at 3 years 4 months of age. During the seizure-free period, she could eat by mouth, but returned to tube-feeding after the relapse of frequent seizures. At 4 years 8 months of age, her height was 89 cm (−3.6 SD), weight 13.8 kg (−1.3 SD), and head circumference 43 cm (−4.7 SD). She was tube-fed, could not sit, and had no jargon. Tonic seizures with extension of the bilateral arms, which was associated with vocalization lasting 60 seconds or less and hyperventilation lasting several minutes, were observed daily.

Individual 2, a 3-year, 1-month-old girl, is a younger sister of individual 1. She was born after a 37-week pregnancy by elective cesarean section due to breech presentation. The Apgar scores at 1 and 5 minutes were both 8. Her birth weight was 2,302 g (−1.2 SD), length 44.8 cm (−1.3 SD), and occipitofrontal circumference 33.7 cm (+0.7 SD). She was admitted to the neonatal intensive care unit with a diagnosis of transient tachypnea of newborn. Nasal continuous positive airway pressure treatment and midazolam (MDL) infusion (0.1 mg/kg/h) was used to control the tachypnea. MDL infusion was terminated at 3 days of age, when upward eye deviation, tonic posturing of the arms, and pedaling movements developed. Intravenous MDL controlled the seizures, but intravenous PB and fosphenytoin with tapering of MDL infusion and oral combination therapy of PB, sodium valproate (VPA), and carbamazepine (CBZ) failed to control apnea and clonic seizures of the left or right extremities. At 2 months of age, she was admitted to our hospital. Interictal EEG showed normal sleep background activity. Video-EEG demonstrated shifting clinical seizures associated with spike-wave discharges independently originating from the left or right hemisphere and migrating to the other hemisphere (Fig. 5B). High-dose PB and KBr decreased the seizure frequencies. Brain MRI at 5 months of age revealed a thin corpus callosum, frontal lobe atrophy, delayed myelination, and arachnoid cyst in the left posterior fossa (Fig. 5E, F). She was seizure-free from 3 months, but clonic seizures of one extremity evolving into other extremities, hyperventilation, and tonic extension of the arms with apnea relapsed at 11 months. At 3 years 1 month of age, her height was 87 cm (−1.7 SD), weight 12.9 kg (−0.2 SD) and head circumference 45.2 cm (−2.0 SD). She had not yet obtained head control. She was also tube-fed and exhibited no babbling. On a daily basis, clonic seizures originated from one extremity, which evolved to other extremities and lasted 60 seconds or less, with hyperventilation lasting several minutes, and vocalization followed by tonic extension of the bilateral arms that was occasionally associated with apnea.

*Individual 3*

This 1-year, 11-month-old Malaysian boy is the first child of non-consanguineous parents. Family history was not contributory He was born after 38 weeks of gestation without asphyxia. His birth weight was 3,300 g (50^th^ centile), length 51 cm (50^th^ centile), and occipitofrontal circumference 35 cm (25^th^ centile). Apneic episodes were observed at 6 weeks of age, but were not recognized as seizures; he was only investigated and treated at the age of 2.5 months when the tonic seizures developed. Oral CZP treatment was then started. Brain MRI at 3 months showed mild brain atrophy. Seizures recurred at 5 months of age with different and evolving seizure types, including cyanotic starry-eyed episodes, generalized tonic-clonic, clonic, and frequent focal clonic movements involving different limbs. He was loaded with phenobarbitone and phenytoin, required MDL infusion, and was ventilated for 3 days for worsening seizure intensity and status epilepticus at 6 months of ages. Interictal EEG showed a diffusely attenuated background with ventilation artifact. He was transferred to our tertiary center for management of refractory seizures at 6.5 months of age. Ictal EEG at 6.5 months of age showed electrographic seizures initially from the right hemisphere, which then migrated to the left hemisphere, with subsequent migration from one hemisphere to the other throughout the recording. His initial development was normal and he gained visual pursuit at 2 months, social smile at 3-4 months, and rolling over at 4 months. However, regression associated with epilepsy was recognized after 5.5 months of age, and he lost the ability to control his head, fix and follow objects, smile, vocalize, have purposeful limb movements, and roll over. His seizures slowly improved after starting on a ketogenic diet (KD) and oral levetiracetam (LEV) at 6.5 months of age. By 7.5 months, there was a significant reduction of seizures (> 90%) that manifested as brief, with a blank stare, deviation of eyes to one side, and tonic posturing. Subsequently, there were only intermittent, brief episodes of fever-provoked seizures. His oral LEV was stopped at 9 months of age, and he was treated with only KD. At 1 year and 11 months of age, his growth centiles were 84 cm height (25^th^ centile), 12 kg weight (25^th^-50^th^ centile), and 46 cm head circumference (3^rd^ centile). His seizure control remained good with KD alone, and he had been seizure-free from 20 months of age. He showed encouraging neurodevelopmental progress. By 9 months, he regained his ability to fixate, smile responsively, and roll over. He started to regain some head control again by 11 months of age and was cooing by 15 months. By 18 months, he was able to momentarily sit unsupported and had more vocalization. He continued to make progress, and at 23 months he began to crawl, sit unaided, grasp objects, finger-feed, and exhibit more tuneful vocalization. Clinically, he had an intermittent convergent squint and was much less hypotonic. There were no behavioral concerns and no feeding difficulties, and his general health was good.

*Individual 4*

This 20-year-old Japanese girl is the third child of non-consanguineous parents. Her mother experienced seizures after falling from a roof at 2 years of age, and was treated with drugs for 1 year. She was born after 37 weeks of gestation without asphyxia. Her Apgar scores at 1 and 5 minutes were 9, respectively. Her birth weight was 2,855 g (−0.2 SD), with an occipitofrontal circumference of 33 cm (−0.1 SD). Cyanosis and upward eye deviation during sleep were recognized one day after birth, then clonic-tonic seizures developed. Seizures were refractory to anti-epileptic drugs, and multiple seizure symptoms were observed, including upward eye deviation, tongue spasm, tonic posturing of the unilateral extremities, and tonic-clonic seizure at 6 months of age. Her development was delayed, and she gained visual pursuit and rolling over at 1 and 2 years of age, respectively. Interictal EEG at 10 years of age showed sharp waves over the left central and frontal regions, with a slow background activity of 5 Hz. Brain MRI at 10 years revealed subdural hygroma, bilateral hippocampal atrophy with high signal intensity on FLAIR image, mild atrophy of the cerebellum, and delayed myelination of the temporal lobe. These findings were still observed at 20 years of age, and progressive atrophy of the cerebellar hemisphere and vermis was evident (Fig. 5G-J). She is unable to sit unassisted or speak meaningful words.


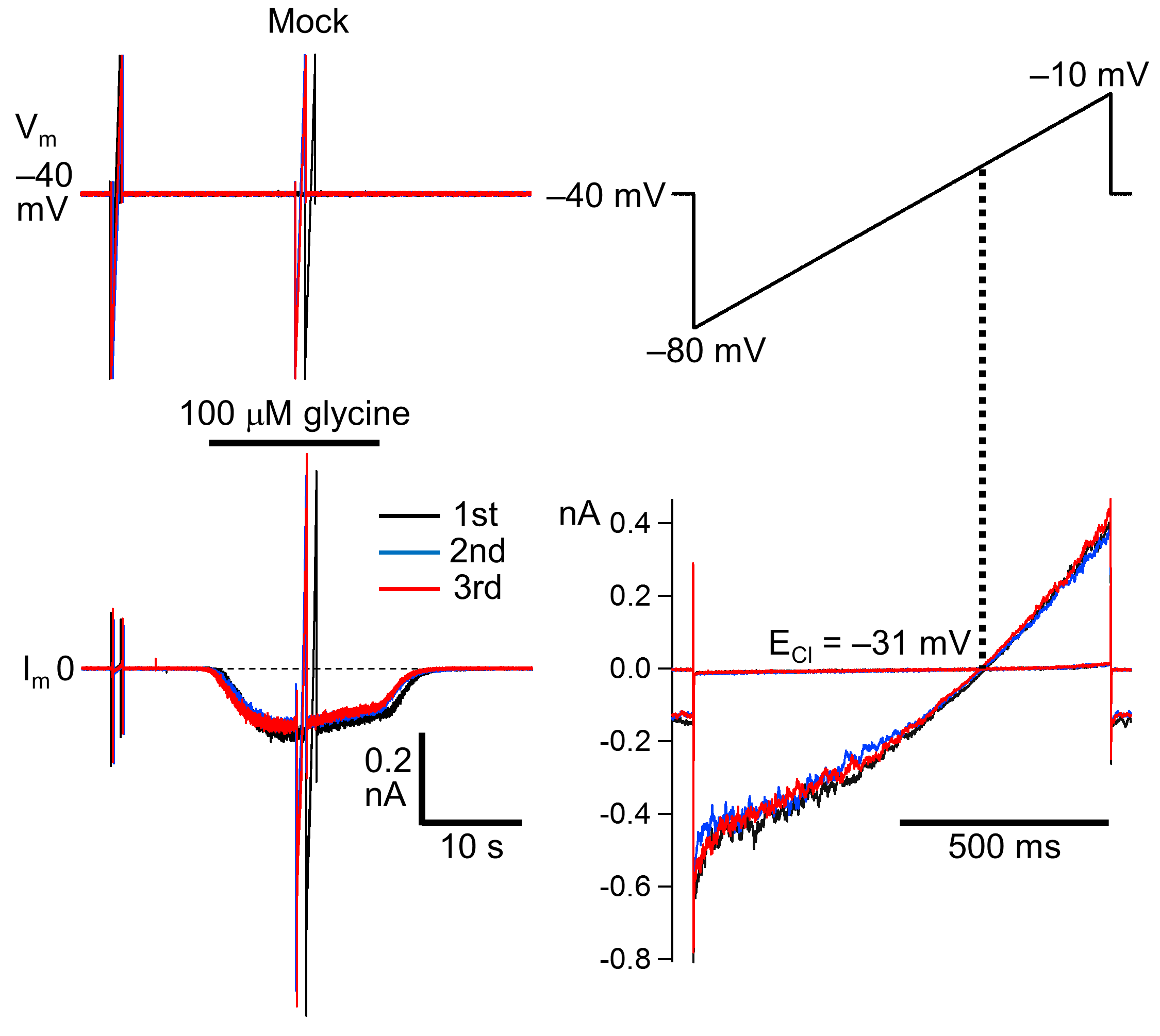


**Supplementary Figure S1**

Representative traces of three successive recordings of glycine-induced current humps and current responses to voltage ramps under the gramicidin-perforated voltage-clamp condition in Mock-transfected HEK293-GlyRα1 cells. The recorded cell was the same as in Fig. 2A, leftmost panel. The left panels indicate superimposed membrane voltage (V_m_, upper) and current (I_m_, lower) traces of three successive recordings. The right panels show the expanded traces of a voltage ramp (upper) and superimposed current responses to voltage ramps (lower). The holding voltage (V_H_) was −40 mV. Two 1-s voltage ramps from −80 to −10 mV were applied before and during bath application of 100 μM glycine for each recording. Recordings were made at 3 min intervals. The 1st record is shown in black, the 2nd in blue and the 3rd in red, respectively. The horizontal dotted line in lower left panel indicates the 0 current level, and the vertical dotted line in right panel indicates the E_Cl_ level. Note almost constant amplitude of glycine-induced current humps and ramp currents during successive recordings. Moreover, the current levels immediately before and after a ramp current during a glycine-induced current hump were almost unchanged, and therefore the time course of glycine-induced current humps was not affected by ramp responses (lower left; see also Fig. 2A). This confirmed that the net Cl^−^ flux across the cell membrane during a ramp response did not significantly alter the intracellular Cl^−^ concentration ([Cl^−^]_i_), i.e. E_Cl_. On the other hand, the original [Cl^−^]_i_ before glycine application might have been altered to some extent after the end of a glycine-induced current hump, according to the total amount of Cl^−^ entry or exit during the hump. Nevertheless, the almost constant current response to repetitive glycine application strongly indicates that [Cl^−^]_i_ was restored to the original level during the application interval. (Otherwise, the amplitude of the hump should have gradually reduced and the measured E_Cl_ should have approached V_H_ = −40 mV during repetitive measurements. We discarded the data when the variation of three successive E_Cl_ values did not converge within ±1 mV, as described in Materials and Methods.) The possible [Cl^−^]_i_ change during a glycine-induced current hump, if any, must be larger in cells with the original E_Cl_ more different from V_H_ = −40 mV. Because the cells showing the measured E_Cl_ more positive or negative than V_H_ should have had the original E_Cl_ more positive or negative than the measured E_Cl_, respectively, the distributions of the original E_Cl_ might have been wider than those of the measured E_Cl_ in our data (Fig. 2B and C). The variation in E_Cl_ in Mock-transfected cells (Fig. 2B, Mock) would reflect different expression levels of various endogenous Cl^−^ transporters in individual cells. In KCC2-transfected cells, the expression level of KCC2 would also contribute to the variation.

**
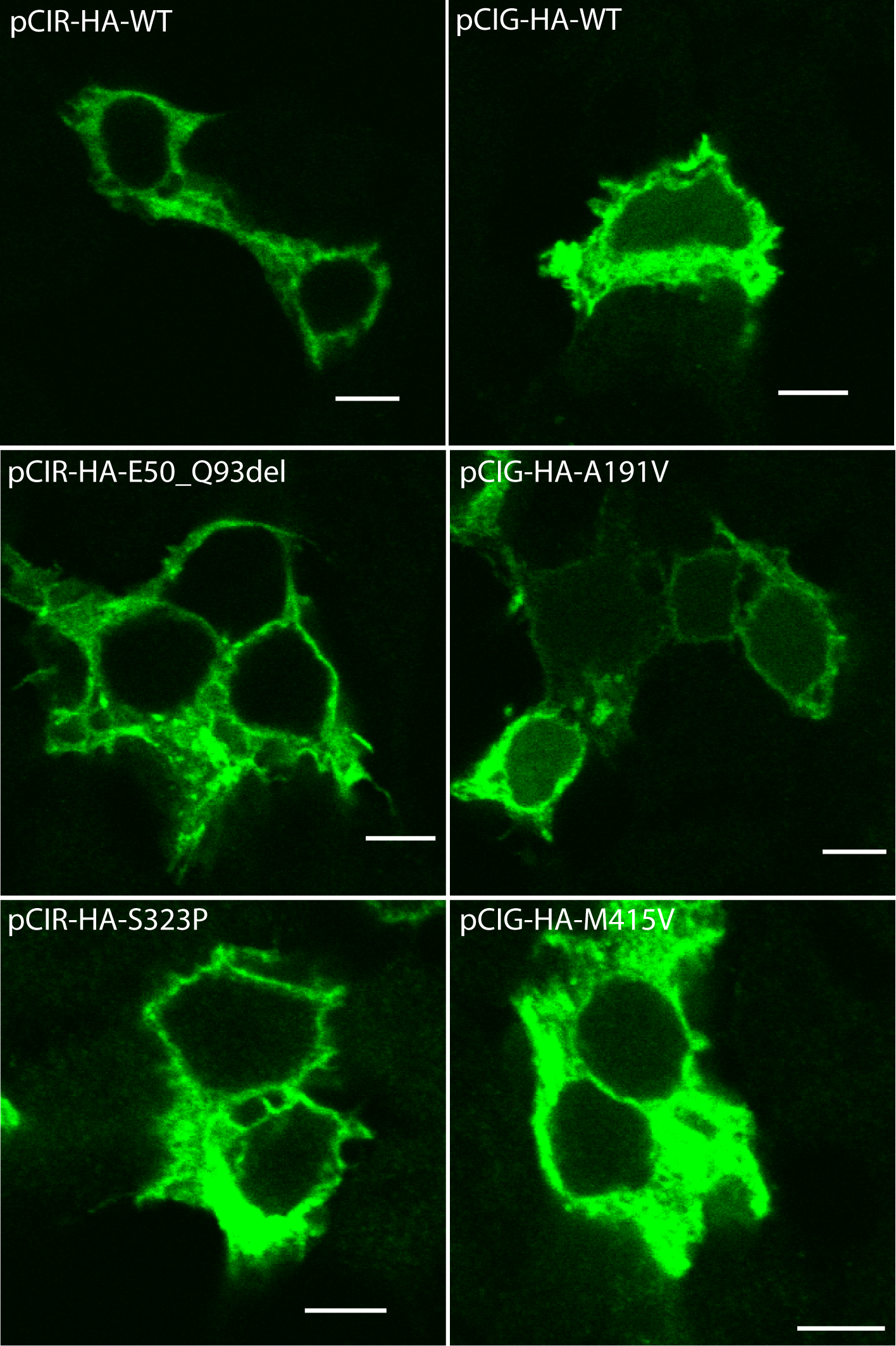
**

**Supplementary Figure S2**

Cellular distribution of individual KCC2 mutants in transfected HEK293 cells. Confocal immunofluorescence images of the cells transfected with individual vectors used in Fig. 3 are shown. Anti-HA antibodies (1:1000; BioLegend) were used as the primary antibody to detect the HA tags on all types of KCC2 above. Anti-GFP and -RFP antibodies were not included here. Expression patterns of KCC2 (green) were similar in WT- and individual mutant-expressing cells. Fluorescence of nuclear-localized EGFP expressed by pCIG vector was vanished by fixation and not detectable in this condition. Scale bars represent 10 µm.

**Table S1.** Summary of exome sequencing performance

|  | Ethnic | Samples | Mean coverage^a^ | %_bases  _above 5× | %_bases  _above 10× | Rare variants  (filter-passed)^b^ | Rare nonsynonymous variants^c^ | Common  in two siblings |
| --- | --- | --- | --- | --- | --- | --- | --- | --- |
| Family 1 | Japanese | Individual 1 | 127.53 | 97.3 | 96.6 | 461 | 309 | 122 |
|  | Japanese | Individual 2 | 141.22 | 97.4 | 96.8 | 458 | 272 |  |
|  |  |  |  |  |  |  |  |  |
| Family 2 | Malaysian | Individual 3 | 76.95 | 97.1 | 95.7 | 945 | 583^d^ | - |

^a^Coverage data were calculated against protein coding sequences of RefSeq genes.

^b^Variants that passed GATK hand-filtering, below minor allele frequencies of 1% in dbSNP135 data, not found in more than five of our in-house 575 control exomes.

^c^Protein-altering and splicing-affecting variants (including synonymous variants located within 2 bp from an exon-intron border).

**Table S2. Pathogenicity prediction of the *SLC12A5* mutations**

| Individual | cDNA change | Amino acid change | Inheritance | SIFT | Polyphen2 | Mutation  Taster | dbSNP138 | EVS database | In-house  database | EXaC database |
| --- | --- | --- | --- | --- | --- | --- | --- | --- | --- | --- |
| 1 & 2 | c.279+1G>C | p.Glu50_Gln93del | Maternal | N/A | N/A | N/A | - | - | 0/575 | - |
| 1 & 2 | c.572C>T | p.Ala191Val | Paternal | 0.00 | Probably damaging  0.999 | Disease-causing  0.999 | - | - | 0/575 | - |
| 3 | c.967T>C | p.Ser323Pro | Paternal | 0.02 | Benign  0.003 | Disease-causing  0.991 | - | - | 0/575 | - |
| 3 | c.1243A>G | p.Met415Val | Maternal | 0.00 | Possibly damaging  0.728 | Disease-causing  0.999 | - | - | 0/575 | - |
| 4 | c.953G>C | p.Trp318Ser | Paternal | 0.00 | Benign  0.058 | Disease-causing  0.999 | - | - | 0/575 | - |
| 4 | c.2242_2244del | p.Ser748del | Maternal | N/A | N/A | Disease-causing  0.999 | - | - | 0/575 | - |

N/A, not applicable. *SLC12A5* Variants were annotated based on transcript variant 2 (NM_020708.4).

SIFT (http://sift.jcvi.org/): scores < 0.05% predict intolerant substitutions.

PolyPhen-2 (http://genetics.bwh.harvard.edu/pph2/): HumVar scores were evaluated as 0.000 (most probably benign) to 1.000 (most probably damaging).

Mutation Taster (http://www.mutationtaster.org/): alterations were classified as disease-causing or polymorphisms.

EVS, Exome Variant Server (<http://evs.gs.washington.edu/EVS/>).　EXac database (<http://exac.broadinstitute.org/>).

**Table S3.** Reverse transcriptase-PCR conditions and primer sequences

| **Exons** | **Product Size (bp)** | **Forward primer (5’>3’)** | **Reverse primer (5’>3’)** |
| --- | --- | --- | --- |
| Ex2-7 | 609 | GGGAAAGGAGTATGATGGCAAGAACA | GTGAGCACACAGGTGCCGTAAACAC |

The PCR reaction comprised 45 cycles of the following conditions:

98°C for 10 sec, 74°C for 30 sec, 5 cycles

98°C for 10 sec, 72°C for 30 sec, 5 cycles

98°C for 10 sec, 70°C for 30 sec, 5 cycles

98°C for 10 sec, 68°C for 30 sec, 30 cycles

68°C for 7 min
